# Case Fatality Rate of COVID-19 and its Relationship to Sociodemographic Characteristics in Ecuador, 2020

**DOI:** 10.3389/ijph.2022.1604768

**Published:** 2022-09-06

**Authors:** Karla Margarita Flores Sacoto, Galo Antonio Sanchez Del Hierro, Felipe Gonzalo Moreno-Piedrahita Hernández, Jose Xavier Jarrin Estupiñan

**Affiliations:** ^1^ CERPOP, UMR 1295, Inserm, Université de Toulouse - Université Paul Sabatier - Toulouse III, Toulouse, France; ^2^ Facultad de Medicina Pontificia Universidad Católica del Ecuador, Quito, Ecuador; ^3^ Centro de Investigación para la Salud en Latino América (CISeAL), Quito, Ecuador

**Keywords:** mortality, COVID-19, SARS-CoV-2, socio-demographic factors, epidemiological model, Ecuador

## Abstract

**Objective:** To analyze sociodemographic factors’ influence on COVID-19 case fatality rate (CFR) in Ecuador on a subnational level.

**Methods:** Publicly available register-based observational study. A retrospective cohort of COVID-19 infections between epidemiological weeks 8–53 in the Ecuadorian public healthcare system was determined from available records. Statistical analyses were conducted to evaluate CFR trends according to factors such as sex, age, location, and healthcare provider.

**Results:** Overall CFR was 9.4%; by canton, median CFR was 5.2%, with some cantons with much higher rates, like Santa Elena (39.1%). Overall CFR decreased during the period, from 16.6% (week 8) to 2.63% (week 53). Being in a rural area was an independent protective factor. Patients over 65 had a hazard ratio of 11.38 (95% CI [11.05, 11.72]). Sex, ethnicity, and treatment from public facilities were also associated with death risk.

**Conclusion:** CFR is a proxy indicator of COVID-19 impact in Ecuador, and this location-based analysis provides new information on the disease’s specific impact subnationally. Overall COVID-19 CFR during the entire period was high, suggesting the need to improve COVID-19 care in Ecuador.

## Highlights

• The CFR is a proxy indicator of COVID-19 impact in Ecuador, and location-based analysis provides new information on the specific impact of this disease on a subnational level and its relationship to sociodemographic factors. The overall CFR of COVID-19 during the entire period was high, emphasizing the need to improve COVID-19 care in Ecuador.
• The risk factors associated with higher CFR were being male, aged over 65 years, living in an urban area, and receiving treatment from the public healthcare system.

## Introduction

Severe acute respiratory syndrome coronavirus 2 (SARS-CoV-2) was identified as the cause of COVID-19, which was declared a pandemic on 11 March 2020 [1]. The virus was first identified in Wuhan in Hubei province, China, in December 2019 and subsequently become a major concern worldwide [2]. COVID-19 is a rapidly spreading respiratory disease whose etiological agent is an RNA virus related to the coronavirus family [3]. SARS-CoV-2 has a dynamic reproduction number (R) depending on the variant and population factors [4, 5]. The most frequent symptoms are fever, cough, and dyspnea [6]. According to collected data, as of 31 December 2021, there were 287,316,743 cumulative total cases worldwide and 5,433,370 total deaths [7], with an estimated global case fatality rate (CFR) of approximately 1.89% [7]. Further, the epidemiological presentation of COVID-19 is dynamic and changes over time; for example, at the beginning of the pandemic, the most affected region was Asia. However, Latin America has also had high infection rates, in which socioeconomic factors likely play an important role; for instance, inequality, overcrowding, and poor access to water and sanitation may raise COVID-19 contagion risk, as well as disease severity and mortality [8].

CFR refers to the proportion of people who die from a certain disease out of the total number of people diagnosed with that disease during a specific period; it is used to measure a disease’s impact on the population and has been applied as an epidemiological indicator for COVID-19 [9]. Ecuador has the eighth highest prevalence of COVID-19 in Latin America [10] and a very high CFR. On 29 February 2020, Ecuador reported its first case of COVID-19, becoming the third country in the region to confirm its presence [11]. The first provinces to report positive cases were located in Ecuador’s coastal region, where many severely ill patients were diagnosed. Subsequently, the infection rate rapidly increased, mostly in the provinces of Pichincha (Andean region), Guayas, and Manabí (both coastal region). By the end of 2020, 251,765 cases of COVID-19 had been identified in Ecuador, and in all 24 Ecuadorian provinces, individuals were diagnosed with severe cases of pneumonia due to SARS-CoV-2. This study reviews the Ecuadorian experience of COVID-19 during 2020 with an emphasis on fatalities by location. It contributes to filling the gap of published studies on the subnational country-level and on the relationship of demographic characteristics to COVID-19 fatality rates.

## Methods

### Design

This is a retrospective cohort study of COVID-19 cases reported to the Ministry of Health (MoH) based on records from the Ecuadorian public healthcare system. We used the Reporting of Studies Conducted using Observational Routinely collected Data (RECORD) guidelines for data analysis and results description.

### Setting

Ecuador’s healthcare system comprises public and private healthcare facilities. The provision of health services by the public sector includes MoH and the social security system. The public sector represents 86% of the country’s health services, while the rest is covered by the private sector. As part of public health reporting system, individual case records are created at the point of health delivery and sent daily to the MoH to provide data for epidemiological indicators, an activity that was reinforced at the beginning of the pandemic. The records are managed through the MoH’s National Office of Epidemiological Surveillance and a governmental coordination that provides preparation, supervision, and management. If a patient dies, a death certificate is generated at the time of death by the Ecuadorian Institute of Statistics and Census (INEC in Spanish). The dataset for COVID-19 cases and deaths during 2020 was created in January 2021 and sent by email to the principal author after administrative approval.

### Data Sources and Access

The study population was determined from the dataset provided by the MoH and INEC. The U07.1 *COVID-19, virus identified* or U07.2 *COVID-19, virus not identified* were classified as “COVID-19 positive” to be included in our study sample. The International Classification of Diseases 10th Revision declares that U07.2 is assigned when COVID-19 has been diagnosed clinically or epidemiologically but lab results are inconclusive or not available, or testing is not performed. We included only cases that occurred between epidemiological weeks 8 and 53 (from 21 February to 31 December 2020) according to the Ecuadorian epidemiological calendar.

All analyses were based on secondary data from the MoH and INEC, which constitute public data that can be requested from either office. Each COVID-19 case was identified with a unique code number and anonymized before we reviewed it. Cleaning and handling of the database was done in Excel.

### Variables

The variables considered were sex, age, ethnicity, comorbidities, disease severity (outpatient or hospital care, death), location (canton, province, and region), housing characteristics (wall, ceiling and floor material, indoor bathroom) and epidemiological week of diagnosis. Sex was based on case data (male/female), and ethnicity was classified as white, black, mestizo, Montubio (mestizo from the Ecuadorian coast), indigenous, or unknown. Death was based on the registration by INEC, as recorded in the dataset. The presence of comorbidities was a binary variable (yes/no).

### Statistical Analysis

CFR (i.e., number of COVID-19 infections that led to death out of the total number of COVID-19 cases for each category) was estimated for sex, age group, epidemiological week, report month, and location (canton, province, and region). To compare between groups, we created smoothing series plots to analyze CFR trends according to each group. Spearman correlation analysis was used to estimate the impact of local caseload in contrast to the geographic location CFR and sociodemographic characteristics.

### Survival Analyses

We conducted survival analyses to assess the risk of death over the course of the study period and the relative impact of its correlated factors. We performed a descriptive analysis using selected variables. For the survival analysis, the dependent variable was time (measured in days), defined as the difference between the date of death during follow-up and the date of first report.

We carried out univariate and multivariate analysis with Cox regression models to estimate each independent factor’s hazard ratio (HR) and its 95% confidence interval.

For the sociodemographic analysis, the dependent variable was mortality rate (per 100,000), defined as the number of deaths from COVID-19 divided by the total population of the same year. The selection of the independent sociodemographic variables was based on correlation between CFR and the data from national surveys from INEC categorized into national and subnational levels.

All analyses were performed with SPSS version 22 and R software version 4.0.0 and its interface RStudio version 1.4.1106 [36, 37].

## Results

**FIGURE 1:**
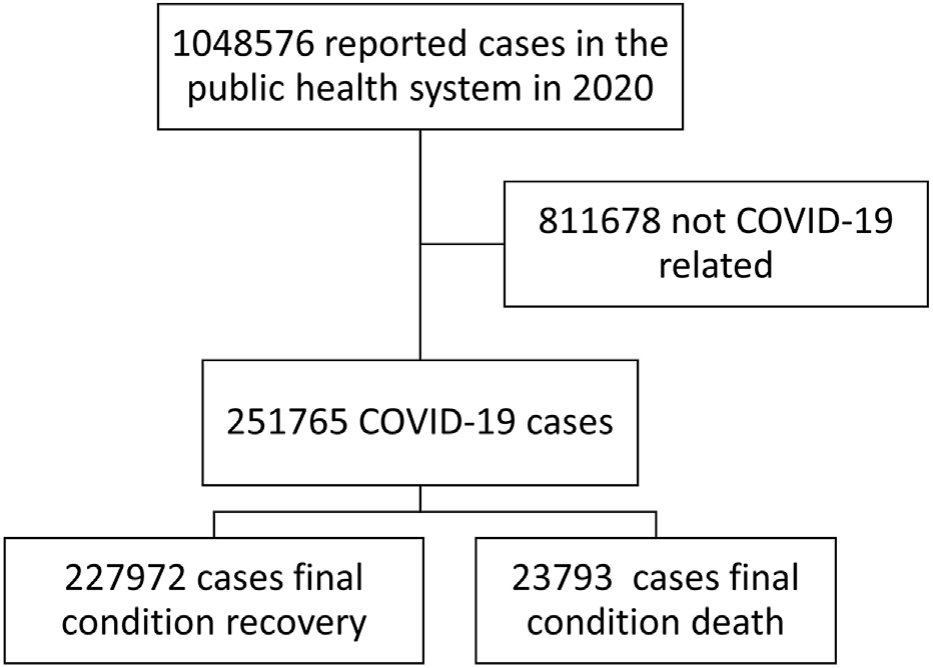
Case selection flowchart from the reported cases, Ecuador.2020.

From epidemiological week 8 to week 53, the MoH received 1,048,576 reports of COVID-19 related cases from 24 provinces (total Ecuadorian population by December 2020: 17,468,736). Of these, 251,765 (22.5%) had positive PCR test results, and 23,793 (9.4%) had died (Figure 1).

Regarding the 251,765 COVID-19 cases, the number of deaths of men (66.3% of total deaths) exceeded those of women during the study period (Table 1). In terms of ethnicity, the highest percentage corresponded to mestizo group (81,7%) followed by indigenous people (1.9%). Black people had the highest CFR (9.9%) of analyzed ethnicity categories.

**TABLE 1:** Characterization of COVID-19-related cases, Ecuador. 2020.

Variable	Death outcome		Total COVID-19 cases n (%)	
	Yes n (%)	No n (%)		OR (95% CI)
Sex*				
Male	15,702 (11.7)	118,414 (88.3)	134 116 (53.2)	1.8 (1.74, 1.85)
Female	8,091 (6.9)	109,558 (93.1)	117,649 (46.7)	
Age*				
<19 years	165 (1.2)	13,282 (98.8)	13,447 (5.3)	11.3 (11.05, 11.7)
20–39 years	793 (0.8)	98,967 (99.2)	99,760 (39.6)	
40–59 years	5,175 (6.5)	74,726 (93.5)	79,901 (31.7)	
60–79 years	12,363 (26.8)	33,770 (73.2)	46,133 (18.3)	
80+ years	5,292 (42.3)	7,227 (57.7)	12,519 (5)	
Ethnicity*				
Black	104 (9.9)	952 (90.2)	1,056 (0.4)	1.26 (1.01, 1.016)
White	39 (9.1)	391 (90.9)	430 (0.2)	
Indigenous	215 (4.5)	4,534 (95.5)	4,749 (1.9)	
Mestizo	6,572 (3.2)	199,146 (96.8)	205,718 (81.7)	
Montubio	121 (7.4)	1,520 (92.6)	1,641 (0.7)	
Unknown	16,742 (43.8)	21,429 (56.1)	38,171 (15.1)	
Type of health care*,^a^				
Outpatient	793 (0.3)	209,335 (99.6)	210,128 (88.7)	1.27 (1.26, 1.28)
Hospitalized	4,894 (21.7)	17,620 (78.26)	22,514 (9.5)	
ICU	3,239 (76.1)	1,017 (23.9)	4,256 (1.8)	
Area*				
Urban	22,618 (11.3)	176,767 (88.7)	199, 385 (79.2)	5.13 (5.2, 5.9)
Rural	1,175 (2.2)	51,205 (97.8)	52,380 (20.8)	
Comorbidity*,^a^				
Yes	2,630 (16)	13,760 (84)	16,390 (6.9)	6.14 (5.8, 6.4)
No	6,296 (3)	202,511 (97)	208,807 (88.1)	
Not known	0	11,701	11 701 (4.9)	
Type of institution*				
Public	19,320 (10)	173,722 (90)	193,042 (76.7)	3.9 (3.7, 4.1)
Private	1,542 (2.8)	54,250 (97.2)	55,792 (22.2)	
Unknown	2,931 (100)	0	2,931 (1.2)	
Nationality*				
Foreign	651 (39.7)	1,636 (60.3)	2,287 (0.9)	3.9 (3.5, 4.2)
Ecuadorian	23,142 (10.2)	226,336 (89.8)	24,9478 (99.1)	

*p < 0.001.

a 14,867 cases with no information reported.

The highest CFR was among individuals aged 65+ (29.1%) followed by the group 50–64 years old (12%). Interestingly, the third-highest CFR by age group corresponded to those under 1 year old (9.5%). Men aged 65+ has the highest CFR when the data were individualized (35.8%) (Table 2).

**TABLE 2:** Case fatality rate by age and sex, Ecuador. 2020.

		Male	CFR %	Female	CFR %
		No. of deaths (%)		No. of deaths (%)	
Age group	0	24 (51)	9.45	23 (48.9)	9.75
	1–4	18 (64.2)	3.33	10 (35.7)	2.03
	5–9	14 (56)	1.60	11 (44)	1.17
	10–14	14 (58.3)	0.87	10 (41.7)	0.59
	15–19	21 (51.2)	0.66	20 (48.8)	0.55
	20–49	1,605 (69.2)	2.19	715 (30.8)	1.03
	50–64	4,523 (69)	16.11	2,028 (30.9)	8.45
	65+	9,479 (64.2)	35.88	5,273 (35.7)	31.16

We observed that CFR exhibited a distinct geographic pattern. CFR by canton (*N* = 221) was 9.4 (mdn = 5.2; min = 0; max = 32.4), and the mean of confirmed cases by canton was 1,138.76. The provinces with the highest CFR were Santa Elena (16.21), Los Ríos (9.2), and Guayas (9.2), all of which are located in the coastal region. The highest morbidity rates were in the Galapagos (4,997.33) and in the Amazon provinces of Orellana (2,968.97) and Pastaza (2,445.1). The highest crude mortality rates corresponded to the provinces of Santa Elena (145.38), Santo Domingo (117.18), and El Oro (84.17). The highest CFR was found in the coastal region. A total of 82 (37%) cantons had a CFR above the median, of which 72% corresponded to the coastal region (Table 3).

**TABLE 3:** COVID-19-related cases by region, Ecuador. 2020.

	Case fatality rate (%)**	Morbidity rate (per 100,000)**	Mortality rate (per 100,000)*
AMAZON (41)	1.95 (SD ± 2.4)	1,778.6 (SD ± 1,462.8)	32.1 (SD ± 41.23)
COAST (84)	8.69 (SD ± 8.8)	741.6 (SD ± 555.2)	64.36** (SD ± 85.49)
GALAPAGOS (3)	1.8 (SD ± 2.2)	4,997.3 (SD ± 4,494.6)	54.2 (SD ± 35.27)
ANDEAN (93)	3.69 (SD ± 3.9)	1,207.5 (SD ± 829.7)	45.24*** (SD ± 61.71)

KOLMOGOROV–SMIRNOV TEST <0.001; KRUSKAL–WALLIS TEST.

*p = 0.09

**p < 0.001.

*N* = 221 Cantons.

**FIGURE 2:**
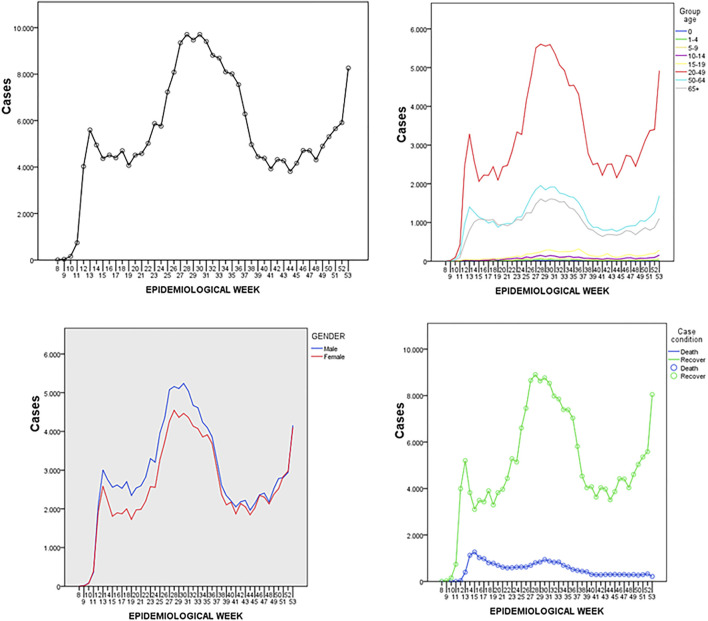
Timeline of 251,765 COVID-19 related cases stratified by sex and age during epidemiological weeks 8–53, Ecuador. 2020.

The highest number of deaths occurred between epidemiological weeks 14 and 19, with a CFR of 22.14%. The highest number of cases were reported between epidemiological weeks 25 and 36; the case peak was reached during epidemiological week 28, when 9,706 cases were registered (Figure 2).

The analysis by geographic region showed a relationship between different periods of the epidemic and the number of local cases. The coastal region had the highest number of deaths during 2020, reaching 1,105 deaths during epidemiological week 15 in Guayas province. This region was also the first to reach its attack rate peak during week 13 with 4,881 cases. The Andean region reached its attack rate peak during week 31 with 7,587 cases, while the Galapagos region had the lowest number of cases overall and reached its peak with 334 cases during week 47; it never surpassed 400 weekly cases during the entire analyzed period (Figure 2).

Ecuador showed a bimodal distribution of cases: an initial growth trend occurred until epidemiological week 15, followed by a second growth trend starting on week 23 that peaked between weeks 25 and 31. Although lethality was highest during week 16, which correlates to the coastal region’s CFR, a later lower peak in week 30 is correlated to the Andean region’s CFR.

The Cox regression model for COVID-19-related CFR in Ecuador considered personal characteristics as well as healthcare facility and location. Age showed the greatest individual HR among those analyzed, wherein cases involving those aged 65+ years corresponded to a 7.87 (95% CI [7.66, 8.08]) increased likelihood of death. This was followed by being male, which had an HR of 1.54 (95% CI [1.49, 1.57]), and being treated in a public facility, with an HR of 1.81 (95% CI [1.55, 2.12]), both of which were significant.

Ecuadorians were less likely to die (HR: 0.38; 95% CI [0.351, 0.41]) than foreigners, as were individuals from rural areas (HR: 0.26; 95% CI [0.246, 0.277]). According to the survival analysis, mortality decreased from August to November 2020 and increased in April and July; Spearman’s rank correlation was calculated to assess the relationship between CFR and sociodemographic factors by canton. There was a positive correlation between CFR and population density, *r* (217) = 0.47, *p* < 0.001; ethnicity (black), *r* (217) = 0.040, *p* < 0.001; ethnicity (Montubio), *r* (217) = 0.36, *p* < 0.001; and good wall material, *r* (217) = 0.34, *p* < 0.001. There was a negative correlation between CFR and rurality, *r* (217) = −0.37, *p* < 0.001; illiteracy, r (217) = -0.14, *p* < 0.05; and no indoor bathroom, *r* (217) = −0.31, *p* < 0.001.

## Discussion

One of the most effective ways to determine the impact of COVID-19 is by examining mortality, which varies widely among countries and depends on the healthcare system response regarding effectiveness of testing policies, healthcare system capacity, and efficacy of response to health emergencies [12]. As reported by Li et al. early in the pandemic, the fatality rate among 1,994 patients with COVID-19 was 5% (95% CI [0.01, 0.11]) [13].

The current study found that after a period of early growth, the national CFR of COVID-19-related cases in Ecuador remained high after the first wave; it decreased only after the end of the second wave. This trend was correlated with various factors related to age, sex, geographic area, and healthcare system, which changed throughout its evolution in response to available information and availability of diagnostic tests that changed from passive to active surveillance which might have impacted on CFR estimations.

The worldwide mortality rate of COVID-19 is 3%, and Salzberger et al. found a CFR of 1.38% with their model [14]. However, this rate varies between countries (e.g., China: 2.3, South Korea: 2.3, Italy: 13.1), as it depends on average age of the population, age distribution, and the healthcare system’s capacity for diagnosis and epidemiological surveillance [15]. The overall CFR calculated in Ecuador (9.4%) through December 2020 is substantially higher than that in other countries of the same region, as well as global CFR [15]. The downtrend is also similar to what most prior studies have indicated: in China, Italy, Spain, and Brazil, mortality decreased in the last period of 2020. Nevertheless, COVID-19 fatality rates are difficult to assess with certainty; data from China, the United Kingdom, and Italy indicate a death rate of approximately 0.7%–1.3% [15–17].

In the current study, when data were classified by age group, the CFR in Ecuador had the same pattern for individuals aged 1–19 years as those observed in other countries [15–17]. Nevertheless, rates were higher in Ecuador among men aged 50 or older and in those less than 1 year old. Although the reason for this high rate in the youngest age group is unknown, some studies have raised potential explanations, including a higher incidence of comorbidities and delaying visits to healthcare clinics [5, 6, 15, 16].

Further, Dong et al. found that children have a clinical progression and disease severity different from adults: 90% experienced mild or moderate disease, and those who developed severe disease had comorbidities that increased the mortality rate [23, 24].

Higher COVID-19 mortality risk is related to age; in China, the mortality rate was less than 0.5% in patients younger than 50 years old and increased to 16% in patients older than 80 [19, 20]. Patients over 60 show higher susceptibility to life-threatening complications from COVID-19 [21, 22].

Although the decline of the CFR over time may correspond to improved understanding of the disease and early use of certain drugs, as much as Ecuador showed a decline in mortality, it remained at a high level in December 2020. Further, case distribution by sex differed considerably, with a higher infection rate in men. The cause of this apparent lower infection risk in women needs further investigation to better understand and improve protective measures.

The demographic characteristics of the Ecuadorian population differ from those in other countries. In 2020, approximately 8% of the Ecuadorian population was aged 65 years or older [18]. COVID-19 has been shown to have higher rates of mortality in older patients in Ecuador. In terms of sex, epidemiological analyses have shown that men have a higher fatality rate than women [13], but the reason behind this phenomenon requires further investigation. In the survival analysis, we found an association between sociodemographic factors and COVID-19 mortality. As in other studies, in Ecuador, being male and older corresponded to a higher adjusted HR of death.

In our study, black people were more likely to die in estimated models than other ethnicities. These findings may reflect socioeconomic vulnerabilities to which a certain population is susceptible, which affect life conditions, lifestyles, and accessibility to healthcare, all of which influence overall health in addition to COVID-19 outcomes. In this case, the population may have worse access to healthcare and to prompt diagnosis, which may reflect unequal access to diagnosis and treatment, which would lead to more accurate reporting and thus a higher mortality rate among this group as compared to the mestizo population.

The CFR is not a constant epidemiological measure but varies between populations and over time and is influenced by external factors like the environment, treatment, and quality of the healthcare system [25]. Reporting global COVID-19 CFR involves taking multiple factors into consideration: 1) diagnostic capacity due to insufficient laboratory testing for COVID-19 patients and 2) reluctance of some COVID-19 patients to report their illness to the healthcare system. As such, the real CFR is difficult to obtain, and data collection affects the fatality rate calculation [35] Ecuador’s CFR is under the risk of underreporting as health system capacity of diagnosis and treatment changed during time and case definition went from passive to active surveillance.

In Italy, mortality was higher in patients aged 70–89 [26], while in Ecuador, the highest mortality rate was for approximately the same age but differed for individuals under 1 year old (CFR: 9%). This high rate of mortality could be related to the surveillance strategy used in Ecuador, as COVID-19 tests were targeted toward symptomatic patients, and thus patients with mild symptoms may not have been identified [27].

Regarding preventive measures carried out, Bates et al. applied a binomial regression analysis and found that unemployed individuals, stay-at-home spouses, manual laborers, and individuals with an elementary school-level education had lower levels of knowledge regarding COVID-19, which supports the correlation we found between illiteracy and CFR by canton and may be related to access to official COVID-19 information [28].

During the early period of COVID-19 in Ecuador, Ortiz-Prado et al. found that men were at a higher risk of dying from COVID-19, as were older individuals and those with comorbidities, which is consistent with the sex- and age-related results in the current study [29].

The spread of COVID-19 in Latin America and the Caribbean region was correlated with socioeconomic factors [30]; there was a significant correlation between COVID-19 and urban poverty rate (*r* = −0.77; *p* = 0.01) and urban extreme poverty rate (*r* = −0.79; *p* = 0.01) [31]. In our study we found the same relation between CFR, socioeconomic factors, and poverty level, suggesting that inequality is related to the spread of COVID-19 and death risk.

High poverty rates were associated with higher rates of COVID-19 infection, which may be explained by the relation of more people having informal jobs in countries with lower Gini index values and higher increase to viral exposure [31]. It appears that there is a relationship between inequality and population heterogeneity; Biggs et al. studied the impact of income level, inequality, and poverty on health status and its dependance on wealth distribution, which is demonstrated on infectious diseases like COVID-19 [38].

Thus, incidence of infectious disease is related to socioeconomic, environmental, and ecological factors, and, in terms of COVID-19, poverty has shown to be a potential risk factor [32, 33]. The current study found that education level, family income, occupation, ethnicity, and number of people in a household are socioeconomic factors related to hospitalization for COVID-19.

### Conclusion

The results from this study show that, in 2020, Ecuador had high morbidity and mortality rates of older patients with confirmed COVID-19 infection and that men were more affected than women (1.98:1).

Within Ecuador, COVID-19 deaths were mainly observed among older male patients. The increase in the number of infected patients reflects a lack of medical resources.

However, these data are limited and were derived from the first year of documented COVID-19 cases in Ecuador; more extensive and larger-scale studies are required to identify factors related to fatality rate.

From a scientific perspective, the subnational differences on CFR discussed highlight the need for transparency in policies for reporting cases; clear reporting of the factors used to calculate CFRs and the age, sex, and location of affected individuals are crucial when comparing COVID-19 cases and mortality rates between different regions. These data are important for governments and non-governmental organizations to identify characteristics associated with high fatality rates and accordingly develop specific measures to prevent or intervene during this health crisis and lessen the consequences related to the burden of COVID-19.

The multilevel relationships between sociodemographic factors and mortality caused by COVID-19 indicated by this study can guide policymakers in their efforts to control and prevent future COVID-19 outbreaks [34, 35].
